# Direct observation of Thermomyces lanuginosus lipase diffusional states by Single Particle Tracking and their remodeling by mutations and inhibition

**DOI:** 10.1038/s41598-019-52539-1

**Published:** 2019-11-07

**Authors:** Søren S.-R. Bohr, Philip M. Lund, Amalie S. Kallenbach, Henrik Pinholt, Johannes Thomsen, Lars Iversen, Allan Svendsen, Sune M. Christensen, Nikos S. Hatzakis

**Affiliations:** 1 Department of Chemistry & Nanoscience Center, Thorvaldsensvej 40, University of Copenhagen, Frederiksberg C, 1871 Denmark; 2 NovoNordisk center for protein research, Novo Nordisk Foundation Centre for Protein Research, University of Copenhagen, Blegdamsvej 3B, 2200 Copenhagen, Denmark; 3 Novozymes A/S, Krogshøjsvej 36, DK 2880 Bagværd, Denmark

**Keywords:** Single-molecule biophysics, Biocatalysis

## Abstract

Lipases are interfacially activated enzymes that catalyze the hydrolysis of ester bonds and constitute prime candidates for industrial and biotechnological applications ranging from detergent industry, to chiral organic synthesis. As a result, there is an incentive to understand the mechanisms underlying lipase activity at the molecular level, so as to be able to design new lipase variants with tailor-made functionalities. Our understanding of lipase function primarily relies on bulk assay averaging the behavior of a high number of enzymes masking structural dynamics and functional heterogeneities. Recent advances in single molecule techniques based on fluorogenic substrate analogues revealed the existence of lipase functional states, and furthermore so how they are remodeled by regulatory cues. Single particle studies of lipases on the other hand directly observed diffusional heterogeneities and suggested lipases to operate in two different modes. Here to decipher how mutations in the lid region controls Thermomyces lanuginosus lipase (TLL) diffusion and function we employed a Single Particle Tracking (SPT) assay to directly observe the spatiotemporal localization of TLL and rationally designed mutants on native substrate surfaces. Parallel imaging of thousands of individual TLL enzymes and HMM analysis allowed us to observe and quantify the diffusion, abundance and microscopic transition rates between three linearly interconverting diffusional states for each lipase. We proposed a model that correlate diffusion with function that allowed us to predict that lipase regulation, via mutations in lid region or product inhibition, primarily operates via biasing transitions to the active states.

## Introduction

Lipases such as the one from *Thermomyces lanuginosus* (TLL) are degrading fat and the tight regulation of their activity is central for controlling a plethora of vital biological processes. Their use in a spectrum of industrial applications including detergent industry and chiral organic synthesis^1–5^, makes them an ideal target for design of tailor-made function to meet increasing industrial needs^6,7^. Lipases in general display very low activity for monomeric water-soluble substrates. This is because the active site of most lipases is covered by a lid, that upon interaction with a water-lipid interface, is displaced exposing the active site and activating the lipase^8,9^. Changes in lid structure has been shown to significantly alter lid dynamics and the function of lipases in general and TLL here^10,11^. A few different TLL variants have been constructed with variations in the residues 71–77. Lipase variant 2 (L2), contains lid like the one of ferulic acid esterase FAEA^12^, a lipase variant known to attain an open lid conformation in solution. L2 is thus hypothesized to have a more open conformation. This mutant has been shown to exhibit much lower activity than native^10,13^, albeit having a more open lid and therefore displaying an interfacially independent activity^10,11^. Additionally, recent MD simulation studies indicate L2 to have the least flexible lid conformation^13^ when compared to native. L3 variant on the other hand is rationally designed to have a hybrid lid, with structural origins from both native TLL and FAEA^12^. Interestingly, this mutant was later found to have a more dynamic lid than native TLL, activate more favorably under conditions mimicking interfacial activation (low polarity solvent)^11^ and, possibly as a corollary of these, attain a slightly higher activity under some conditions^13^.

Current understanding on activity regulation of lipase, and protein in general, primarily relies on crystallographic evidence and studies reporting the average activity of large ensembles of enzymes in solution, by measuring concentration changes over time. Reporting the averaging behavior of a large ensemble of biomolecules often masks protein dynamics and their inherent conformational sampling, all of which are expected to underlie regulation of protein biomolecular recognition and function^14,15^. Single molecule studies allow the direct observation of enzyme conformational sampling and the presence of multiple conformations within the average structure^16–21^. The existence of multiple protein conformations^20^ gives rise to activity fluctuations and the existence of multiple protein functional states, as we and others^22–25^ have shown by single molecule studies (referred to as dynamic disorder)^23,25–29^.

Current single molecule characterization of functional dynamics of proteins and their dependence on regulatory cues, primarily rely on fluorescent methods that report changes in fluorescent properties upon enzymatic reaction, and can be summarized in ones that involve fluorogenic substrates^17,23,26,28,30–32^, fluorogenic cofactors^33^ or FRET^34^ studies. Using fluorogenic substrates and parked beam setups offers studies at the fundamental limit of individual catalytic turnover albeit require sequential low throughput readout. Our recent studies on TLL based on this methodology revealed the existence of discrete functional states that were redistributed by allosteric regulation^23^. Single particle tracking methodologies, on the other hand, allow extraction of diffusional behaviors from individual molecules, yielding critical insights in protein function^35,36^. Such pivotal studies of lipases on native substrate layers provided the first insights on the interaction and diffusional properties of lipases with native substrates and proposed the existence of two diffusional states^37^. Deconvoluting docking interactions as well as the function on native substrates is instrumental for deciphering how mutation in the lid region affect the function of lipases.

Here we used a single particle tracking (SPT) assay to directly observe the temporal trajectories of hundreds of individual enzymes synchronously acting on their native substrates with high temporal resolution. We used the metabolic^26,38^ enzyme, TLL on trimyristin layers, which constitute its native substrate^37,39^. By deploying rationally designed variants with mutations in the lid region^9,10^, we sought to gain key insights on mechanistic details on how lid mutation govern TLL function^37,39^ as well as the link between mobility and function. Quantitative analysis of the kinetics, using HMM analysis, revealed each lipase to reversibly sample 3 linearly inter-converting diffusional states, an arrested, practically immobile one (D = 0.05 µm^2^/s), a state with diffusion slightly smaller compared to lipids (D = 0.1 µm^2^/s), (see Fig. S1 for quantification of D of lipid by SPT) as well as states with either diffusion coefficients similar to lipids or significantly faster than lipids (D = 0.3 µm^2^/s and D = 1 µm^2^/s respectively). Studies on lipase variants with mutations in the lid region, known to control function, allowed us to quantify how energetics and thermodynamics of sampling these states are regulated by mutations and additionally develop a linear model of diffusional states. The observed redistribution of conformational sampling by mutations and product presence, allowed us to provide correlations of sampling between diffusional states to the overall lipase function regulation.

## Results

**Figure 1:**
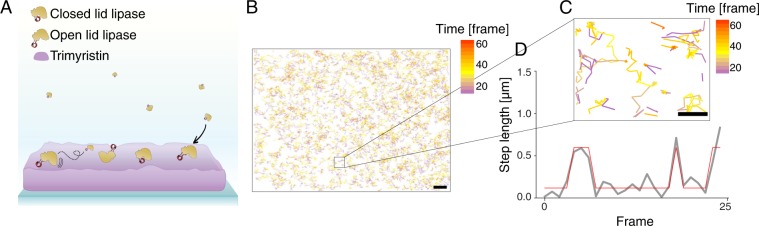
Experimental setup to track individual lipase enzymes on triglyceride substrate layers using Total Internal Reflection microscopy. (**A**) Representation (not to scale) of triglyceride layer labeled with DOPE-ATTO-655 and Alexa Fluor 488 labeled TLL lipases displaying diffusion, multiple potential binding or interaction modes and initial lipase to substrate binding. (**B)** Overlay of typical temporal trajectories of individual lipases displaying lateral diffusion on trimyristin surfaces. Enzyme tracks are color-coded according to observation time. Briefly, the color code display time for a given trajectory in frames observed, purple is after enzyme binding, yellow at intermediate and red after longer observation times. Data from 100 frames are displayed for clarity, Scale bar 5 µm. (**C**) Closeup of traces reveals heterogeneities within diffusional behavior such as total immobilization, periods of slow diffusion or fast diffusion. Color-code as for B. Scale bar 2 µm. (**D**) Typical step length trace of an enzyme displaying reversible transition from initial high mobility to a low mobility state and the corresponding idealized traces found by HMM analysis.

We employed a SPT assay to directly observe the temporal displacement of individual TLL and its dependence on mutations in the lid region and product inhibition. Total Internal Reflection (TIRF) is widely used to record the spatial organization and lateral diffusion of membrane related proteins^40–42^. We ensured a specific directional labeling of lipases by employing single cysteine labeling (D137C) to TLL enzymes and reacting them with Alexa Fluor 488-maleimide. Labeled enzymes were subsequently added to the buffer solution atop a thin layer of the trimyristin substrate surface (Fig. 1A). TIRF imaging allowed the parallelized recording of thousands of TLL trajectories on a trimyristin surface with 97 ms temporal resolution (see Fig. 1B for overlay of >2000 traces, see Fig. S2 average imaging lifetime for each individual enzyme mutant). The high labeling efficiency of 83–86% ensured that the vast majority of enzymes was labeled. Exclusively analyzing data displaying single step bleaching (>95%) ensured single chromophore labeling, monomeric protein imaging and confirms the absence of protein aggregates (see Fig. S2 for individual bleaching steps, and labeling yield). Zooming in on the recorded tracks (Fig. 1C) revealed heterogeneous mobility behaviors, as both freely diffusing, static and molecules temporarily arrested were found.

### Lipase diffusion is correlated to function

**Figure 2:**
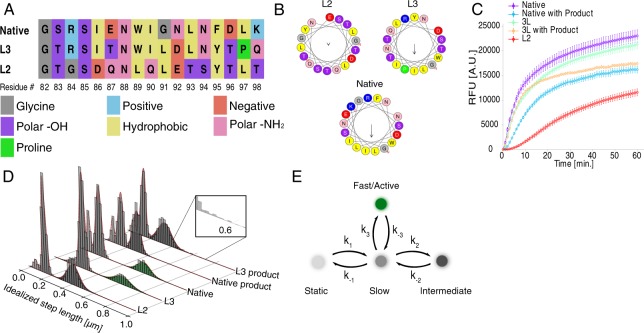
Quantifications of the effect of mutations on TLL activity and diffusional state sampling. (**A**) Sequence alignment of the 3 variants used, color coding denotes the charge or polarity or type of the amino acids. (**B**) Helical wheel representation of all mutations on lid structures generated using HELIQUEST^72^. Native and L3 variants display several larger hydrophobic residues and a relatively high hydrophobic moment compared to L2, which contains less and smaller hydrophobic residues. (**C**) Bulk activity of lipase mutants reveals Native and L3 to display practically identical high activity. L2 displays intermediate activity. Product addition (2% myristic acid) results in inhibition and partial loss of activity. Product inhibition is stronger on native as compared to L3 variant. (**D**) Histograms of step sizes and underlying diffusional states provided by Hidden Markov analysis, see Supplementary Methods M2–M4 for HMM analysis and fitting methodology. Each of the tested lipase variants reversibly transits between 3 diffusional states. The slow and the practically static states (peaks at 0.1 µm and 0.05 µm respectively) appear to be sampled by all variants. Faster state appears to correlate with activity: the higher the activity of the mutant the higher the diffusion coefficient of the fast state. L2 operates via sampling an intermediate mobility state. Product inhibition and mutations lowering activity. (**E**) Proposed model with four underlying states conserved between mutants. Each mutant may sequential sample up to three states within the experimental time frame, the static and slow and either the fast or the intermediate.

Comparison of the data for the selected variants indicates TLL mean diffusion coefficient (D) to correlate with overall bulk activity (see Table 1 and Fig. 2C). Diffusion coefficients were calculated as described in Supplementary Methods M1, values below indicate empirical mean and standard error. The highly active, native and L3, variants display the highest average diffusion (D = 3.5 · 10^−10^ cm^2^/s and 4.7 · 10^−10^ cm^2^/s respectively). L2 variant, with reduced activity, was found to have D = 3.1 · 10^−10^ cm^2^/s. Although the distribution of diffusion coefficients are wide and overlapping, they are significantly different (verified by two-sided Welch’s test, see Supplementary Table S4) and thus elute a link between high mobility and high function, which to be confirmed requires a more quantitative analysis.

**Table 1:** Quantification of average diffusion and binding probabilities for lipases and respective mutants.

Lipase	Total tracks	Average Diffusion 10^−10^ [cm²/s]*	Binding probability
Native	4.804	3.5 ± 1.7	31.6%
Lid mutation 3 (L3)	62.642	4.7 ± 1.1	32.1%
Lid mutation 2 (L2)	44.523	3.1 ± 1.0	51.2%
Native product	12.432	1.7 ± 1.1	31.2%
Lid mutation 3 (L3) product	3.013	2.8 ± 1.3	30.8%
DOPE-ATTO655 (SPT)	2.267	3.7 ± 0.01	na**

^*^Error corresponds to one standard deviation.

^**^Not extractable/ensemble.

We expect the enzymes to undergo some form of Brownian motion, under the assumption that they will be moving as hard spherical particles^43^ embedded within the substrate surface and exposed to solvent drag. A key element of classical diffusion theory is that many models are asymptotically defined^44^ and thus require long trajectories to converge^45,46^. To compensate for the fact that SPT may yield primarily short trajectories and be of stochastic nature, we chose a different approach than the classical mean square displacement, which require long track to converge properly. Instead we used the same method as we published earlier^42^, by analyzing the probability density function for observed step lengths. Calculation of the hydrodynamic radius using Stokes-Einstein theory^43,47^ from particle diffusion coefficient (see Supplementary Method M5), results in radii practically identical for all variants, within error. Assuming spherical particles, we report radii ranging from 1.8 ± 0.6 nm for the slowest variant, to 1.2 ± 0.4 nm for the fastest (see Supplementary Table S5). The found hydrodynamic radii are in great agreement with earlier reported values^48^. The fact that the radii are identical within error for all variants provides limited insights on the potential effect of varying hydration layer^43^ among mutants to the observed diffusional behavior (see Supplementary Table S5). The agreement of the extracted sizes with earlier studies using orthogonal methods, may further validate the Brownian motion hypothesis.

Several control experiments ensured the validity of our readouts. Labeled and non-labeled enzymes displayed the same bulk behavior showing fluorophore labeling not to affect their function^39^ (see Fig. S3). Surfaces retained their structural integrity for the entire experimental time (see Fig. S4). Similarly, SPT measurements on trimyristin layer (with 5 ppm Atto-655 DOPE) revealed lipid diffusion to be constant and independent of lipase addition within the experimental time frame (<5 min, see Fig. S1). These data indicate that lipid hydration or lipase hydrolysis and myristic acid production do not significantly affect trimyristin layer properties within the experimental time frame.

### Effect of lid mutations on lipases diffusional properties

We next quantified how TLL docking and diffusional properties depend on lid mutations that we have recently showed to affect lid dynamics and function (see Fig. 2A,B)^10,11,13^. L2 contains ferulic acid esterase (FAEA) lid, while L3 had a hybrid lid composition of both FAEA and TLL character. Native and L3 variants display several large hydrophobic residues and a relatively high hydrophobic moment compared to L2 which has smaller hydrophobic residues. L2 is expected, and experimentally shown, to primarily sample the open lid configuration albeit display lower activity, where L3 and native on the other hand are found to have similar activities as native and slightly higher different lid dynamics^10,11,13^.

The L2 mutations in the lid region resulted in large increase of the normalized surface recruitment probability P_dock_ when compared to more active variants_._ We calculated this by finding the fraction of enzymes that remain docked for at least 2 frames, P_dock_ = N_dock_/N_total_, where N_dock_ is the number of lipase trajectories that last for at least two frames and N_total_ is the total number of particles detected (including N_dock_ and particles only observable for one frame). Normalization of docking event to the number of recorded traces, by dividing with the total number of particles, excluded potential bias due to varying enzyme concentration. While the method is a qualitative estimate of the true recruitment, some interesting observations were made. L3 and native have similar docking properties P_dock_ ~31% (see Table 1), as expected based on lid properties. L2 displays higher P_dock_, 51%. This is surprising, as due to the absence of a hydrophobic wedge on the lid, one would expect a lower binding to the lipid surface^49^. The lack of hydrophobic wedge on L2, results in a shift of the lid equilibrium towards an open configuration^11^ as recently described^9,50,51^ causing consequently the increased capacity of L2 to hydrolyze substrates below the CMC^10^. This equilibrium shift consequently exposes a large hydrophobic patch of the protein to the water solvent^10^. One possible explanation is that this exposure may destabilize the soluble enzyme and lead to increased binding, shown here as an increased P_dock_. The increased P_dock_ of L2 may indicate increased affinity of L2 for the substrate but verifying this falls out of the scope of this paper. The increased docking of L2 is interesting, as it has been hypothesized earlier, that the reduced activity of L2 was due to lower binding^10,11,13^. Our single particle approach points towards another mechanism, where we hypothesize it may be interlinked with the observed reduced mobility.

### Analysis of step length distributions reveals distinct diffusional behaviors redistributed by lid mutations

To investigate for the existence of multiple mobility behaviors, as hinted by visual inspection of the traces in Fig. 1C, the distribution of step lengths for each lipase variant was fit with respectively 1 to 4 gamma distributions and evaluated the optimum using BIC values (see Supplementary Table 1 for BIC values). The analysis revealed, that all mutants and conditions are best described by 3 underlying states with characteristic mobility, which was then applied in the following segmentation by HMM. This analysis of the step size^24,52–56^ (see Fig. 1D for idealized trace and Fig. S5 for more traces) yielded three clear distributions of step lengths, Fig. 2D, that correspond to three states with varying diffusion coefficients for each mutant and condition. Careful inspection of the distributions revealed, that while each enzyme appears to sample three populations, the summary of states sampled by all enzymes is four. The fidelity of the data treatment methodology was confirmed by simulated single molecule trajectories (see Fig. S6), using both step lengths and HMM analysis. The fact that 3 states pertain to all tested lipase variants (see Fig. 2D) and regulatory conditions indicates this to be a pervasive phenotype underlying their behavior.

**Figure 3:**
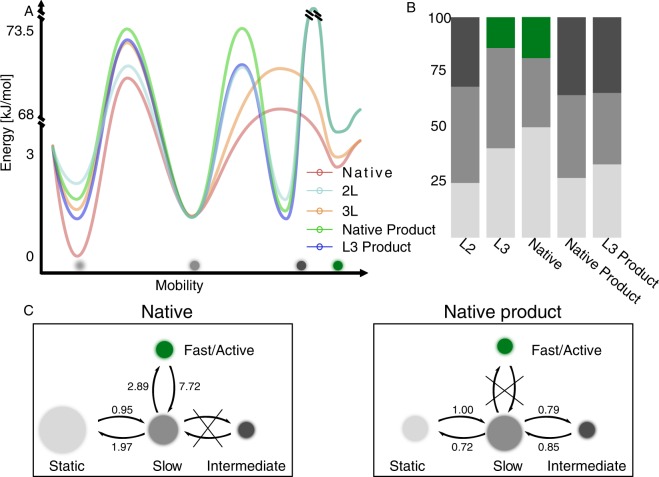
Representation of 2D Energy landscape of TLL diffusional states sampling and its biasing by regulatory cues and lid mutations. (**A**) Cartoon representation of free energy landscape based on functional states, for the native enzyme and L2 shows the three distinct sampled states and the energy barrier between them, as well as the forbidden states within our experimental time frame. (see Table S3, Fig. S10 for all rates). **(B**) State occupancies for all lipase mutants and their dependence on environmental regulatory cues and mutations. The fast mode is only observed in the highly active native and L3 variants. Intermediate activity variant L2 or product inhibitions, operate via eliminating sampling of the fast diffusional and sampling of an intermediate state instead. (**C**) Model with native TLL diffusional states displaying the microscopic transition rates and its redistribution by product inhibition (see Fig. S12 for all conditions). Product Inhibition of TLL operates by rerouting conformational sampling pathways.

We developed a model to account for TLL diffusional states where each of the diffusional state would correspond to a state with different activity, Fig. 2C. Each lipase variant reversibly samples 3 diffusional states *en route* to catalysis. Out of these states there is a slow and a practically immobile one (see Fig. S7 for irreversible immobilized particles), (with mean step sizes 0.1 µm and 0.05 µm respectively), and a diffusional state with higher mobility, the occupancy and diffusion coefficient of which varies depending on the lid mutation. The higher activity mutants (native and L3) spent 19% and 14% of their time (see also Fig. 3) in a fast-diffusional state (step size 0.6 µm), while the intermediate activity variant (L2) displays ~31% probability to sample an intermediate diffusional state (step sizes ~0.3 µm). The fast diffusional state is ~2x faster than lipid diffusion, (see Figs 2D, S1), in agreement with evidence on charged fatty acids produced during catalysis to propel the enzyme^38^. We attribute therefore the fast state to a highly active state, where hydrolysis and product productions appears to propel the enzyme. The fact that bulk activity measurements here and earlier^10,11^ display L3 and native to have similar activity and, furthermore so, higher than L2 further supports this hypothesis. The higher presence of anomalous diffusion parameter alpha >1 (see Fig. S8), indicating some form of active transport similar to the “ballistic” mode reported earlier^38^ for acetylcholinesterase and urease, further support the fast diffusional states to correlate with higher activity (see Fig. S8 for rest of data). The intermediate diffusion state, sampled by L2 on the other hand, has diffusion similar to that of phospholipid (see Table 1). This state, while active, would display very slow product formation not propelling the enzyme. This could originate from the less hydrophobic lid of L2 that could result in improper orientation of the enzyme on the lipid interface as suggested earlier^13^ or imperfect active site organization. The major difference between the intermediate activity variant L2 and the highly active variants (native and L3) is the sampling of the intermediate and the highly active states respectively, indicates a correlation of diffusion to activity; the higher the mean step length of the state, the higher the activity. The slow diffusional as well as the static state on the other hand seems to be inactive. The fact that across multiple experiments L3 rarely samples the static – attributed to practically inactive state – indicates a functional advantage compared to native variant.

### Mechanistic insights on state redistribution by thermodynamic and kinetic analysis

The single particle trajectories and the corresponding transition density plots (TDP’s, see Fig. S9) revealed a well-defined, linear pathway of sampling lipase diffusional states (Fig. 2E) where the tested lipases sequentially sample adjacent states, while transition between non-adjacent states seems prohibited. TDP’s allow visualization of state transitions, and thereby provides information on the sequence of the transitions. Analysis of the TDP’s allowed us to extract the dwell time and the microscopic transition rates for all pairs of transitions as we did recently^20^. Using a combination of k-means clustering and two dimensional gaussian mixture model (see Fig. S10 and Supplementary Note M2) allowed us to calculate the ΔG and energy barriers for all diffusional states sampling (see Table 2, Supplementary Tables 2 and 3) for well separated transitions (see Supplementary Method M2 for comments on separation and overlapping clusters). The highly active mutants (L3 and native) directly transit from the slow to the fast-diffusive state. Interestingly the native variant had a slightly lower energy barrier compared to L3 for accessing the highly diffusive state (70.35 and 71.7 KJ/mole respectively) and a lower energy barrier for returning to the intermediate sate (67.9 and 68.7 KJ/mole respectively). As expected, a clear difference is observed for lid mutants, also indicated by earlier studies using MD simulations^13^, where it is suggested that while the lid only tends to open when in lipid contact, variants may exhibit different orientations on the surface. While our data does indicate a significant difference between mutants, we cannot resolve the exact binding or interaction. Both native and L3 mutants have return rate (k_−3_) to the intermediate state that is significantly larger than (k_3_) for native and L3 (by ~2.65 and ~3.3 fold) rendering the sampling of the highly active state transient and short lived. Earlier coarse grained simulation studies^13^ showed L3 to have a highly dynamic lid which in agreement with the dynamic sampling of the highly active open state observe here.

**Table 2:** Thermodynamic and kinetic characterization.

Lipase	Transition rates [s^−1^]					
	k_1_	k_−1_	k_2_	k_−2_	k_3_	k_−3_
L2	2.43 ± 0.014	1.38 ± 0.010	1.59 ± 0.007	2.33 ± 0.011		
L3	1.30 ± 0.023	1.16 ± 0.013			1.67 ± 0.006	5.64 ± 0.017
Native	0.95 ± 0.040	1.97 ± 0.095			2.89 ± 0.041	7.72 ± 0.082
Native product	1.00 ± 0.032	0.72 ± 0.039	0.79 ± 0.023	0.85 ± 0.037		
L3 product	0.97 ± 0.12	1.00 ± 0.10	1.46 ± 0.071	1.34 ± 0.06		

^*^Error corresponds to one standard deviation.

L2 variant on the other hand displays alternative sampling pathway. It does not sample the fast diffusive state (see Figs 2D, S11) in agreement with its low activity measured here (Fig. 2C) and earlier^10,11^. It displays an apparent equilibrium constant of ~1.8 fold for transiting from static to slow state and ~1.4 for transiting to the slow from intermediate, resulting in the slow state be the most thermodynamically stable one. Earlier modeling and functional studies suggested L2 to display increased likelihood of sampling the open lid state, albeit to display low or no activity^10,11^. The 30% likelihood of sampling the intermediate states and the practically 0% likelihood of sampling the highly diffusing state fully agree with these measurements and further support intermediate state to correspond to an intermediate activity state and fast states to active state. The low activity of the L2 variant may is thus due to the prohibited sampling of the fast diffusing active state rather than decreased binding to trimyristin surface.

### Product inhibition diminishes the fast diffusional state

A basic assumption of our model is that diffusion states correlate with functional states. Under this assumption we would predict that inhibitory interaction of TLL would operate primarily via altering the fast states diffusion coefficient or /and redistribute the equilibrium of sampling that state. To test for this prediction we constructed trimyristin surfaces enriched by 2% mol:mol product (myristic acid) that is reducing lipase activity (see Fig. 2C light blue)^57^. Indeed activity assays in bulk confirmed product addition to reduce the overall activity of native and L3 variants (Fig. 2C). Our single particle readout revealed product addition to the native variant to primarily operate via prohibiting the transition to the highly active state and favoring the previously inaccessible transition to an intermediate diffusive state (see Fig. 3A). This could be state similar to the state sampled by the L2 or with a slight different orientation due to the negative charges originating from released product, also indicated by simulated data^13^. The relative rates and occupancies of sampling the slow and inactive states on the other hand, remained practically unaffected for native variant, albeit they are slightly reduced. Transition rates between the slow and intermediate states is significantly slower as compared to transition between slows and fast state for the non-inhibited enzyme. This indicates product inhibition to also reduce the dynamics of lipase conformational sampling between active and inactive states (see Table 2, Fig. S12 and Supplementary Tables 2 and 3). Inhibition of L3 variant displays overall similar redistribution towards the low diffusing states as for the native variant. Careful inspection of the histograms of step sizes show that inhibited L3 retains a minute sampling of the highly active state a phenotype that may explain the lower product inhibition observed by bulk measurements (see Fig. 2C,D). Interestingly product inhibition for L3 drastically increased the likelihood to sample the, otherwise rarely sampled, static state.

### Remodeling of energy landscape by mutations and regulatory cues

Relative free energies differences between all TLL states were calculated using the individual rates for all pairs of transition (see Supplementary Methods M2, Figs S9 and S10). The combination of free energies differences and activation energies for all pairs of transitions allowed us to visualize the free energy landscape for each mutant and how mutations and regulatory cues remodel it (Fig. 3, Table 2 and Fig. S12). Because the slow state is practically dominantly sampled, and its mean step length is similar, across all variants and conditions its energy can be approximated to be practically identical for all variants and conditions and could thus be taken as a reference point in our energy landscape. Figure 3B provides a full visualization of how the occupancy of each state is dependent on point mutations and regulatory cues. The inhibited L3 enzyme has decreased energy barriers by 1.5 KJ/mol to transit to the intermediate state as compare to the product inhibited native (72.06 ± 0.011 KJ/mol and 73.55 ± 0.07 KJ/mol respectively) and ~1KJ/mol to return to the slow (72.26 ± 0.10 KJ/mol and 73.38 ± 0.11 KJ/mol respectively). This results in a slight free energy stabilization, albeit reduced dynamic sampling of the intermediate state for the L3 inhibited as compare to native inhibited (see Fig. 3A, and Supplementary Tables 2 and 3. We note that the relatively poor separation of TDP clusters for native-inhibited may result in increased errors in extracting rates and energy barriers (see Supplementary Method M2). Mild product inhibition was found to originate by eliminating transition to the highly active state and favoring transition to the intermediate state.

## Discussion

The dynamic sampling of conformational states governs all aspects of protein behavior from folding to function. These conformational states are expected to correspond to distinct functional outcomes^23,28^ however their quantitative characterization is challenging because a large number of single molecules have to be recorded and may require native substrates. The dynamic exploration of conformational and functional states of lipases has been characterized by us and others at the single turnover level^23,26,31,58^, who also quantified their dependence on regulatory cues. Recent SPT studies relying exclusively on active lipases variants acting on trimyristin surfaces, observed the existence of diffusional heterogeneity and partial arrest, that was attributed to product propulsion and more than one binding conformations^37^. The parallelized and high temporal resolution imaging SPT using quantitative (TIRF) microscopy here combined with the mutant comparison revealed a new mechanistic layer on how lid mutations control lipase behavior on native substrates. The detailed statistical and HMM analysis allowed us to identify the existence, and abundance of four distinct underlying diffusional states that correlated to lipase function. We developed a linear model that correlated the diffusional states to functional and thus conformational states, which allowed us to predict that lipase product mediated inhibition operates via biased of conformational sampling and prohibiting transitions to the active states (see Fig. 3A). Quantitative analysis of the microscopic rates of all pairs of transitions allowed the complete thermodynamic and kinetic characterization of the conformational sampling and consequently mapping the multidimensional landscape of the lipase. Regulatory cues or mutations in the lid appear to remodel the landscape allowing previously practically inaccessible transition and/or inhibiting transition.

The direct observation is a significant new addition to insights on the diffusional properties of enzymes, which until recently was done by e.g. NMR^59^, electrophoresis^60^ or even sedimentation^61^. Assuming the diffusion follows Brownian motion, the stokes radius can be derived by following Stokes-Einstein theory, taking into account diffusion coefficient and solvent viscosity. The Stokes radius is informative on both the hydration level and the molecular weight and shape of the protein. Our measurements on diffusion coefficient and the extracted hydrodynamic radius are in great agreement with earlier gyroscopic radius studies^48^ and provide hydration levels similar to earlier published results for similar lipases^47^. The Stokes-Einstein relation may also provide info on lipid structure^47^ and future comparative measurements on additional lipids may shine light to this.

The single particle readout also allowed us to extend beyond the effect of mutations on average activity and deconvolute this effect on the surface recruitment probability as well as the likelihood of sampling the highly active states. L2 mutation, previously thought to have lower activity due to reduced binding, is found to have higher docking on trimyristin surfaces as compared to native enzyme albeit not to sample the highly diffusing states we attribute to function. The low average activity of the L2 variant may thus be due to the prohibited sampling of the fast diffusing active state rather than decreased binding. L3 variants shows limited sampling of the static state. Based on the assumption that the static state is not active, this may indicate L3 has an advantage of avoiding sampling the inactive immobile state. When inhibited by product L3 maintains a low (~1–2%) probability to maintain the highly diffusing state. The fact the L3s lid has decreased hydrophobic moment and consequently increased dynamics^10^, as compared to native, indicates the intricate role of lid mutations, and that deciphering their precise role is crucial for design of new variants.

Recent measurements on these variants in solution, measured the activity of water soluble, free lipase (below CMC) and primarily substrate-bound enzymes (above CMC)^10^. These studies reported L2 to be less active, but able to retain activity without being interfacially activated. L3 and native displayed similar tendencies, both highly dependent on interfacial activation and significantly higher activity as compared with L2 – similarly to our results. Our studies here are optimized to directly observe the enzyme when docked on the natural substrate interface, but general trends for both diffusion as well as activity are in good agreement with earlier published results.

Our data may indicate the presence of a feedback loop type of product mediated inhibitory mechanism. Lipase activity appears to be inhibited by excess product formation and presence in the trimyristin surface. Enzymes remaining in the same area would be downregulated by excess product preventing “over activity” in lipases on their natural substrates. The observed product mediated active transport of the enzyme (alpha values > 1) to new areas may on the other be an efficient way to optimize lipases functional outcome, not unlike the results recently published^38^, but here in a product mediated feedback loop. This chemical diaspora may allow lipases to overcome overcrowding limitations and product-inhibitions and consequently to sense areas where no product is present where they can continuously work, maximizing catalytic proficiency.

The adaptability and flexibility of the reported assay may cover a vast collection of novel mutants and new environmental cues, and easily extend to cover other membrane bound proteins. Furthermore the convenient sample preparation, allows the facile variations in lipid composition. By varying the lipid species (e.g. mixtures of trimyristin and triolein), substrate surfaces with phase separation or hydrophobic defects maybe introduced. These hydrophobic defects would act as binding sites for amphipathic helix insertion^49,62^ and lipase binding in general^36,62,63^, as we and other have shown. Varying lipid chain length may significantly alter the activated lipase activity, as it depends on triglyceride chain length^64^ and exhibits a preference for medium length substrates. Based on these and recent findings, we would expect similar or reduced binding on substrates that are in solid phase^65^, like tripalmitin. Similarly due to reduced activity towards tripalmitin, we would anticipate also a reduced mobility, albeit this remains to be experimentally validated.

Importantly, the presented methodology may allow in the future, simultaneous high throughput measurements of diffusion and enzymatic activity, using pre-fluorescent substrate analogues^66^ thus unifying structure/function measurements at the single molecule level. A better understanding of how mutations and environmental factors remodel the lipase landscape may prove vital in expanding our understanding enzymatic behavior and function and disentangle the molecular mechanistic details that underlie their regulation. Insights in enzymatic regulation of function and their direct relation to structure may provide the basis for future design of tailor-made enzymatic functions, where one design new mutations to address specific needs.

## Materials and Methods

### Materials

All used chemicals were of analytical grade and purchased from Sigma-Aldrich (Denmark) unless otherwise stated. Alexa-488 mono-functional maleimide was purchased from Thermo Fisher Scientific, Denmark. Labeled phospholipids, 1,2-Dioleoyl-sn-glycero-3-phosphoethanolamine-ATTO655 (DOPE-ATTO655) were purchased from ATTO-TEC GmbH, Siegen, Germany. 1,2-Di-O-lauryl-rac-glycero-3-(glutaric acid 6-methylresorufin ester) (lipidated resorufin) (Sigma-Aldrich, CAS: 195833-46-6), glyceryl trimyristate (trimyristin) (Sigma-Aldrich, CAS: 555-45-3), Trizma base (TRIS) (Sigma-Aldrich, CAS: 77-86-1), n-hexane (Sigma-Aldrich, CAS: 110-54-3), 96% ethanol (Alere, CAS: 64-17-5), microtiter plates (ThermoFisher, Cat. # 237107).

### Protein engineering

Protein engineering and purification was performed as described earlier^10^.

### Protein labeling

The free cysteine C137, located on the backside of the active side, was labeled specifically with Alexa Fluor 488 mono-functional maleimide as described by the manufacturer’s protocol (Thermo Fisher Scientific) and separated from free dye as earlier reported^22^. The labeled enzyme was flash frozen via liquid nitrogen and stored at −80 °C until use. All labeled lipase variant was prepared similarly.

### Lipase activity assay

Lipase activity was determined in 50 mM TRIS buffer at pH 7, 8 and 9. The bottom of microtiter plate wells were carefully coated with lipid using a 1:200 molar ratio of lipidated resorufin and trimyristin dissolved in hexane at a concentration of 20 µM and 4 mM, respectively. 100 uL of the hexane solution was carefully pipetted onto the bottom of each well and left to evaporate in a fume hood at a minimum of three hours in dark minimizing the bleaching of lipidated resorufin. A stock solution of 10 mM lipase substrate in 96% ethanol was used. Trimyristin was used in powder form. To run the assay, 200 uL of TRIS buffer with or without lipase at 51 nM was carefully loaded into the wells and the increase in fluorescence intensity in the solution above the lipid layer due to release of resorufin analog was measured every minute for one hour in a plate reader (Tecan Infinite M1000 PRO) with excitation at 530/10 nm, emission at 590/10 nm and gain at 100. The assay was run at 24 °C.

### Total internal reflection fluorescence (TIRF) microscopy

All single particle tracking (SPT) experiments were performed on using a Total Internal Reflection Fluorescence (TIRF) microscope (IX 83, Olympus), using two EMCCD cameras (ImagEM X2, Hamamatsu) and oil immersion 100x objective (UAPON 100XOTIRF, Olympus), resulting in a total pixel width of 160 nm. Alexa-488 and ATTO-655 fluorophores were excited using 488 nm and 640 nm solid state laser lines respectively (Cellsense, Olympus). Imaging of enzymes (Alexa-488) was done using 80 ms exposure time, 300 EM gain and a resulting framerate of 10.1 s^−1^. All experiments were done using the same experimental setup.

### Trimyristin substrate surface preparation

Trimyristin substrate surfaces were made using an in-house developed method, adapted from^37^. Trimyristin were dissolved in toluene to a concentration of 20 g/L (27.66 mM) and mixed with 5 ppm DOPE lipid attached Atto 655 organic fluorophore (DOPE-ATTO655, mol:mol). The lipid solution was spin coated Ø25 round microscopy glass slides at 5000 rpm for 60 s, followed by 1 s pause, follow by 60 s at 5000 rpm. The samples were placed in custom made teflon chambers, and then subjected to high vacuum for at least 5 hours before immediate use. For experiments with product incubation, 2% (mol:mol) myristic acid were added to the trimyristin/DOPE-ATTO655 solution prior to spin casting.

Prior to imaging, 59 µl 50 mM TRIS buffer were added to the chamber, followed by 1 µl labeled enzyme, resulting in a final concentration of ~0.5 nM protein. Solution were allowed to equilibrate for 1 min for image recording.

### Image analysis and single particle tracking (SPT)

Quantitative image analysis was done using a customized version of TrackPy^67,68^ together with in-house developed routines for detailed analysis. In order to test the validity of the software, we initially tested the tracking using simulated data (see Fig. S3). Here we created a video of randomly distributed gaussian intensity spots on a surface with similar noise as the experimental setup and allowed them to move following a single diffusion Brownian model. Inspection of the true step length distribution and the one from the tracking software revealed almost identical results. For experimental data only particles with 15 or more observed locations were used for further analysis. MSD was calculated as described in^69^ and^36^:

$${\rm{MSD}}({\rm{\tau }}={\rm{n}}\ast \Delta {\rm{t}})=\langle {{\rm{r}}}^{2}({\rm{\tau }})\rangle \ast \frac{1}{{\rm{N}}-{\rm{n}}}\ast \mathop{\sum }\limits_{{\rm{i}}=1}^{{\rm{N}}-{\rm{n}}}{({{\rm{x}}}_{{\rm{i}}+{\rm{n}}}-{{\rm{x}}}_{{\rm{i}}})}^{2}+{(({{\rm{y}}}_{{\rm{i}}+{\rm{n}}}-{{\rm{y}}}_{{\rm{i}}}))}^{2}$$

where ∆t is the frame interval, N is the number of total frames and x_i_ and y_i_ are coordinates at t = i. From the MSD, the instantaneous diffusion coefficient can be found together^70^ along with the anomalous diffusion parameter, alpha, found by fitting the first 15 time lags of the MSD by,

$${\rm{MSD}}=4\ast {\rm{D}}\ast {{\rm{t}}}^{{\rm{a}}}$$

For extraction of diffusion coefficients we deployed a method using a simple Brownian diffusion model to describe the data, as we and others have used previously^42,71^, where the probability to a given steplength r is given by,

$${\rm{p}}({\rm{r}},{\rm{t}},{\rm{D}})=\frac{{\rm{r}}}{2{\rm{Dt}}}\ast \exp (-\frac{{{\rm{r}}}^{2}}{4{\rm{Dt}}})$$

where r is the observed steplength, D is the diffusion coefficient (determined using the maximum likelihood approach, see Supplementary Methods M1 for detailed explanation) and t is the time between consecutive steps.

### Data analysis

All data analysis was done using custom made scripts in python. See Supplementary Methods for detailed information regarding Hidden Markov Model (HMM) analysis, Bayesian Information Criterion (BIC), Transition Density Plots (TDP’s) and the resulting state lifetimes, rates and relative energies.

## Supplementary information

Supplementary Material

## Data Availability

All data is available for download upon request.

## Code availability

All code used within can be downloaded at www.hatzakislab.com.
