# Supplementary material for: Direct observation of Thermomyces lanuginosus lipase diffusional states by Single Particle Tracking and their remodeling by mutations and inhibition
Source: Sci Rep. 2019 Nov 7;9:16169. doi: 10.1038/s41598-019-52539-1 (PMC6838188; doi:10.1038/s41598-019-52539-1)
Supplement: Supplementary file 1 — Supplementary Material [file 41598_2019_52539_MOESM1_ESM.pdf]

Supporting information

Direct observation of Thermomyces lanuginosus lipase diffusional states by Single Particle Tracking and their remodeling by mutations and inhibition

Søren Schmidt-Rasmussen Nielsen[1,2], Philip M. Lund[1,2], Amalie S. Kallenbach[1,2], Henrik Pinholt[1,2], Johannes Thomsen[1,2], Lars Iversen[3], Allan Svendsen[3], Sune Christensen[3] and Nikos S. Hatzakis[1,2]*

Author affiliations
[1]Department of Chemistry & Nanoscience Center, Thorvaldsensvej 40, University of Copenhagen, Frederiksberg C, 1871, Denmark. [2]NovoNordisk center for protein research Novo Nordisk Foundation Centre for Protein Research, University of Copenhagen, Blegdamsvej 3B, 2200 Copenhagen, Denmark. [3]Novozymes A/S, Krogshøjsvej 36, DK 2880 Bagværd, Denmark.

Correspondence should be addressed to: hatzakis@nano.ku.dk

Supplementary Methods

M1. Extraction of diffusion coefficient from step length.
To extract diffusion coefficient from single particle tracking data, we applied a simple Brownian diffusion model (sub class of the Gamma distribution). The approach was used to a) predict the most likely diffusion for each individual trajectory (and thus extract a mean diffusion, see Table 1) and b) predict the most likely diffusion coefficient for each state in each trajectory found via HMM analysis. The principle of maximum likelihood was used to estimate D, as it has no need for binning of data and hence avoids the introduction of binning bias. To estimate D, we used the equation below (as we published earlier[1]) to maximize the likelihood given a distribution of observed step lengths, where the probability is given by:

$$p(r,t,D) = \frac{r}{2Dt} * \exp\left(-\frac{r^2}{4Dt}\right)$$

Where r is the observed step length, t is the time interval between steps and D is the corresponding diffusion coefficient.

M2. Determination of functional states and transition rates by Hidden Markov Analysis
Deconvolution of underlying functional states was performed by analysis with a Hidden Markov Model approach, similar to published methodologies[2], on the distribution of observed step lengths for each trace following gamma distributions. Analysis of traces across lipase variants revealed, through BIC score (see Table S1), that a 3-state model was the best description. The analysis was done by fitting the total distribution of observed step lengths for each variant by 1-4 gamma distribution(s) and then comparing the results. All found states, for a given lipase variant, were plotted together in a histogram and fitted with a mixture of three Gaussians (four different populations in total, see Fig. 2).

Each pair of mobility transitions found ("state before" and "state after"), were plotted as Transition Density Plots (TDPs), as shown in Fig. S9, and separated into two clusters per diagonal using a combination of k-means clustering, as reported recently by us[3], and two dimensional gaussian mixture model (four clusters in total). The number of clusters were determined from earlier discussed Gaussian distributions, and corresponds to the three underlying states found for each variant. To exclude severe outliers in each cluster, data points outside a 98 % confidence interval (2.5 sigma) of each cluster center was excluded from further treatment. While this ensures reasonable cluster separation, we note that clusters in close proximity may still suffer from overlapping and that this could influence the kinetic characterization (see Fig. S9-10). Transition rates were determined through fitting a single exponential decay to the lifetime of each state (see Fig. S10 for single exponential fits and Table 2 for transition rates)[2]. From this, a four-state linear model (Fig. 2) was derived using similar methods as recently[2,4].

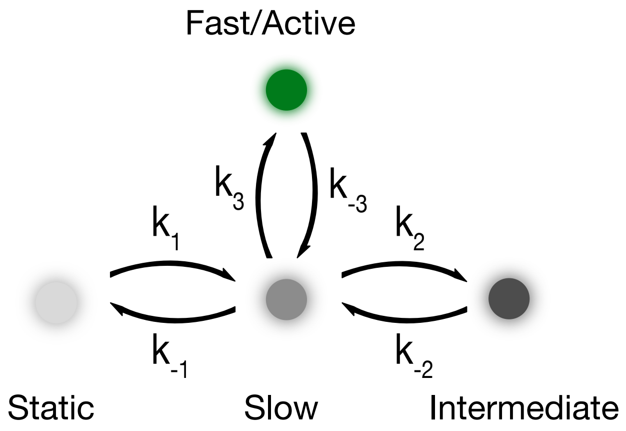

The equilibrium constant between states can be found as,

$$K_{eq} = \frac{k_1}{k_{-1}}$$

From which the relative free energy difference between adjacent states can be calculated,

$$\Delta G = -RTln(K_{eq})$$

where R is the gas constant, T the absolute temperature (here 298 K) and K_{eq} from above. Alternatively, the equilibrium concentrations, and thus state occupancies, can be extracted using the equilibrium constants between states, assuming they sum up to 1 and are interdependent:

$$E1 = \frac{1}{1 + K_1 + K_1 * K_2}$$
$$E2 = \frac{K_1}{1 + K_1 + K_1 * K_2}$$
$$E3 = \frac{K_1 * K_2}{1 + K_1 + K_1 * K_2}$$

where K_n is the equilibrium constant between adjacent states. Note that since all lipase variants display only three out of the four observed global states, the model for each individual variant looks as:

$$E1 \underset{\leftrightharpoons}{\overset{K1}{}} E2 \underset{\leftrightharpoons}{\overset{K2}{}} E3$$

Furthermore, the activation energy (energy barrier), E_a, can be found from transition state theory using the equation:

$$E_a = -RT(\frac{hk_{ij}}{k_B T})$$

where R is the gas constant, T the absolute temperature (298 K), h the Planck constant, k_{ij} the rate from state i to j and k_B is the Boltzmann constant. Note that since all variants display 3 states, the

above can be directly applied - even though the actual mobility of E3 changes between highly active and the less active variants.

M3. Fitting HMM states by Gaussian distributions.
Gaussian distributions were used to describe and deconvolute individual underlying functional states within the observed HMM states (Fig. 2). The fitting was done using the maximum likelihood. Since these methods set to maximize the likelihood function, and the resulting fit is generated from probabilities for each individual data point belonging to a certain Gaussian population, no binning is needed. This was preferred over a tradition non-least-squares approach, as binning may bias the fit and thus elute the true population. All fitting was done using custom made routines in Python.

M4. Bayesian Information Criterion (BIC)
All fits were evaluated using the Bayesian Information Criterion (BIC) a widely employed methodology to avoid overfitting as it punishes over-fitting by penalizing the addition of parameters in relation to the likelihood of the fit[5]. BIC can be calculated as

$$BIC = -2 * \ln(L) + k * \ln(n)$$

where L is the maximum likelihood function for a given dataset and the free parameters, k is the number of parameters included in the model and n is the number of data points. BIC allowed us to identify the optimal number for each individual condition (see Table S1), thus one can utilize this and evaluate what model best describes the data.

M5. Hydrodynamic radius calculations
The hydrodynamic radius of a spherical body undergoing Brownian motion can be calculated from Stokes-Einstein theory[6] using the equation:

$$D = \frac{k_B T}{6\pi\eta r}$$

Where, k_B is the Boltzmann constant, T the temperature in Kelvin, η the dynamic viscosity of the medium and r the Stokes radius (hydrodynamic radius). In this equation the only unknow parameter is the dynamic viscosity of the medium. To utilize the equation, one must assume true Brownian motion, meaning a hard sphere experiencing drag force from a viscous solvent.

As pure trimyristin at 21° degrees does not display a dynamic viscosity[7], we used mobile lipids (see Fig. S1) to estimate the dynamic viscosity of our surfaces, following recently published methods[8]. By estimating the size of DOPE-ATTO655 conjugation to 1.5 nm[9], a resulting viscosity of 5.82 ± 0.016 poise for the slightly hydrated trimyristin was found. From here the hydrodynamic radius of lipases was calculated using Stokes-Einstein. The results can be seen in supplementary table S4.

Supplementary Figures

S1. Lipid diffusion within the trimyristin layer by SPT

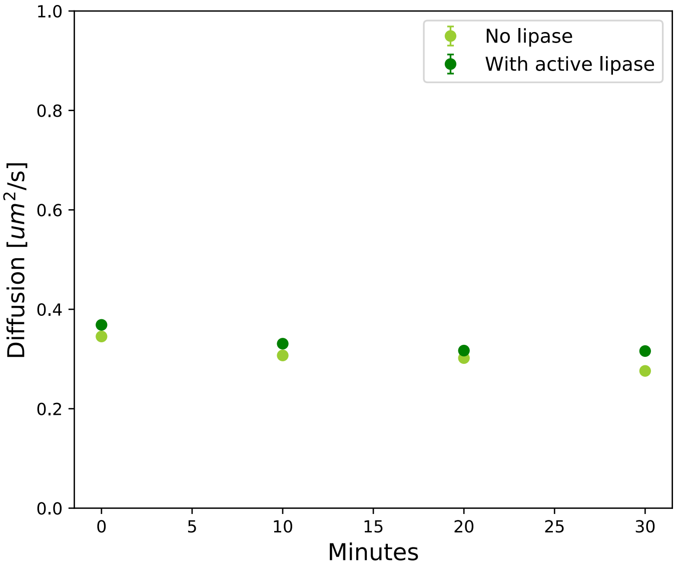

Fig. S1. Lipid diffusion (ATTO-655-DOPE) within the trimyristin surface layer by SPT (1 ppm). A) Single particle tracking of fluorescently tagged DOPE lipids additionally reveals no significant change, again for both samples containing active lipase (dark green) and no lipase (light green). Error bars at least triplicate measurements.

S2. Individual bleaching steps and lifetimes for all lipase types

A

Lipase	Total tracks	Mean lifetime [frames]
L2	44.523	49.5
L3	62.642	42.0
Native	4.804	41.3
Native product	12.432	43.7
L3 product	3.013	42.8

B

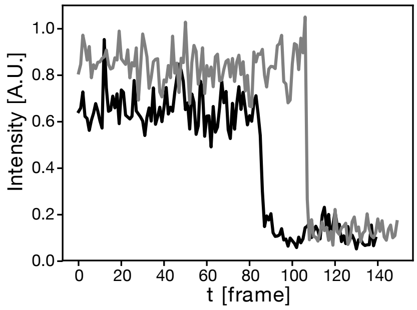

C

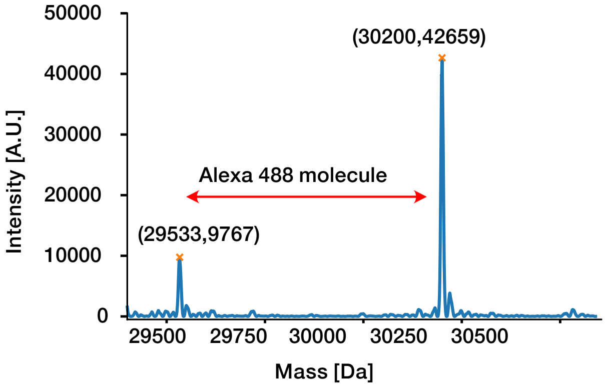

Fig. S2. Tracking characteristics for lipase variants. A) Table of all variant, showing number of tracks and the respective mean lifetimes for all conditions. B) Representative traces of enzymes display bleaching in a single step (normalized to t=0), confirming the docking and consequent bleaching of a single labeled individual lipase. The fact that imaging lifetimes are practically identical for all variants support that signal loss is due to bleaching rather than enzyme dissociation from surface. C) Labeling yield determined by electron spray mass spectrometry (intact mass) to be 83-86 %.

S3. Effect of labeling on enzymatic function

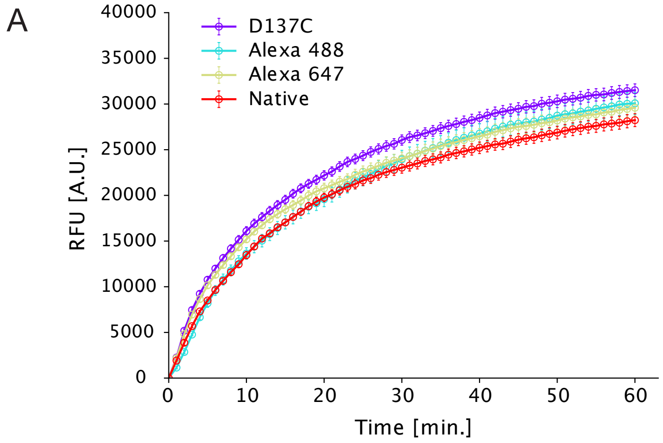

Fig. S3. Effect of labeling on enzymatic function. A) Comparison of enzymatic activity for labeled (yellow and cyan) and unlabeled (red and purple) species display no observable effect of labeling. Red curve shows native variant, purple native with specific cysteine mutation for labeling, cyan display cysteine mutant with Alexa-488 label and yellow the cysteine mutant with an Alexa-647 label.

S4. Surface integrity over time

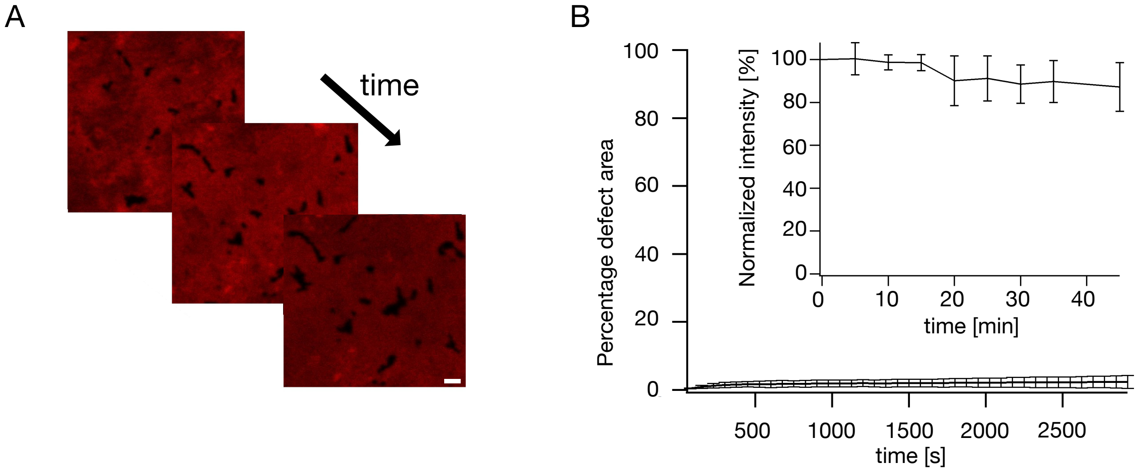

Fig. S4. Surface structural integrity over time after buffer addition, analyzed by confocal fluorescent microscopy. A) Images displaying time correlation of defects in the layer to be very stable over a period of > 40 min. Images are taken at 10 min, 25 min and 40 min respectively. Scale bar 5 µm. B) Area of defects on trimyristin surfaces containing 5 ppm ATTO655-DOPE lipid display little to no change over huge time span. SPT experiments are performed within the first 5 min of exposure to buffer. Error bar indicate triplicate measurements. Inset: Fluorescent intensity as a function of time further confirms the integrity of the substrate surface.

S5. HMM fitted traces

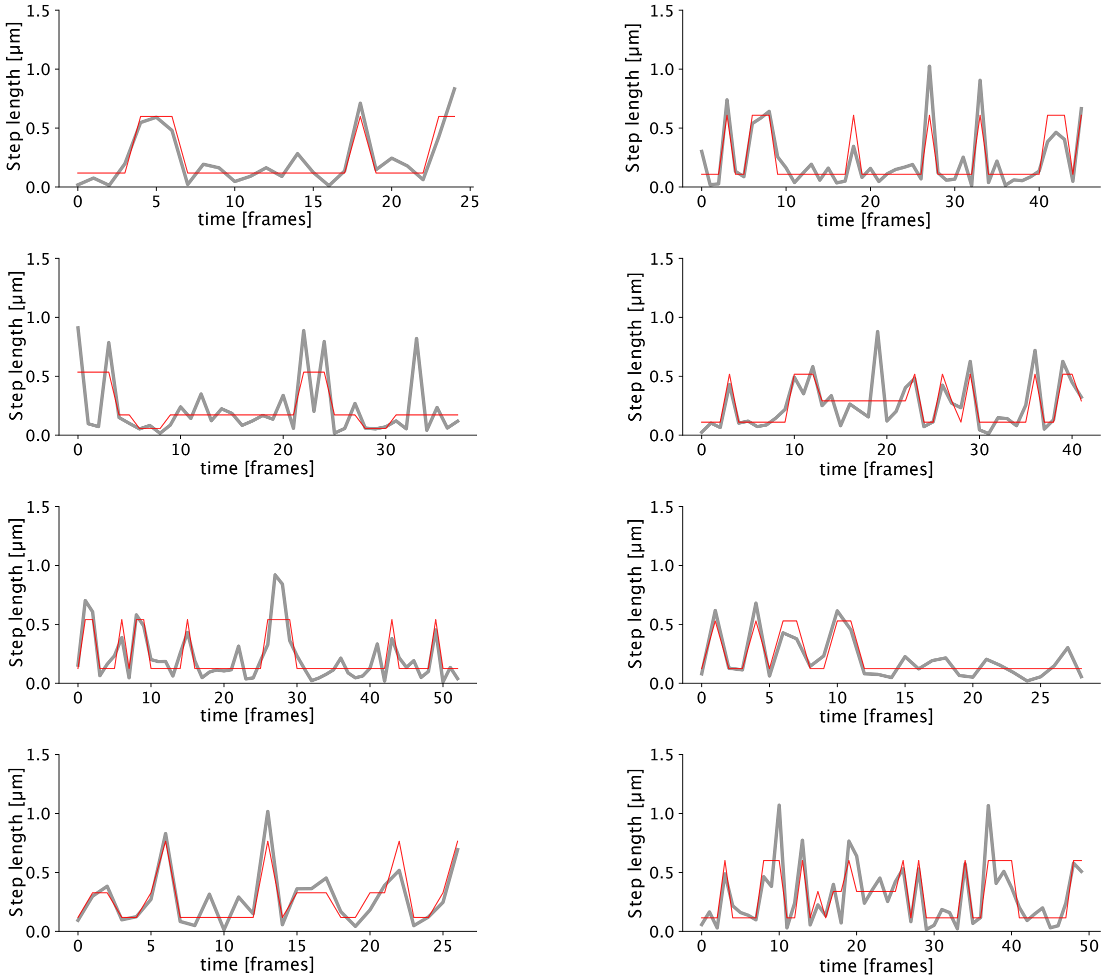

Fig. S5. A collection of representative traces and their HMM idealized states found as described in M3.

S6. Comparison of step lengths from simulation and SPT

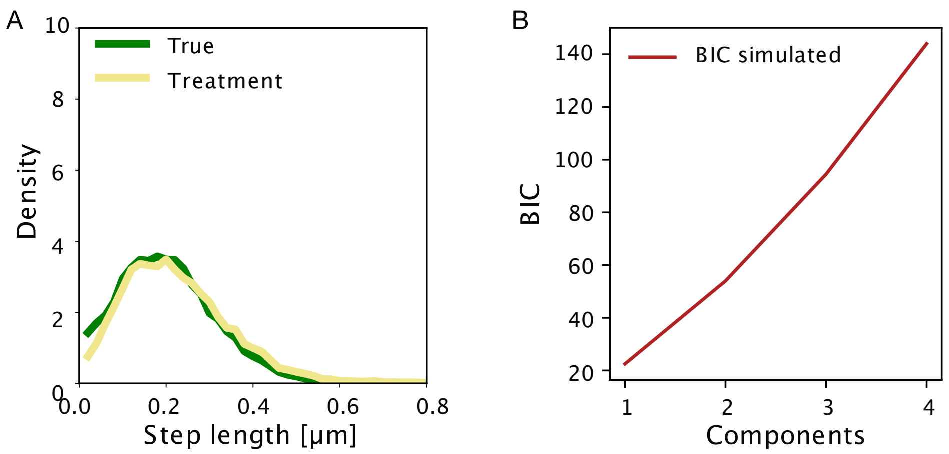

Fig. S6. Comparison of step length from simulation and SPT. A) Step length distributions from simulated data (green) and tracked data (yellow). Data was simulated by making a video of gaussian intensity spots moving on a surface, following a single Brownian diffusion model. Data confirms the software's ability to track single particles on a surface. Noise was introduced to match experimental data. B) BIC values for HMM analysis on the tracked data from A. As expected, the methodology here suggests that a single underlying diffusional state is the best description.

S7. Step length distribution of immobile particles irreversibly bound to the surface

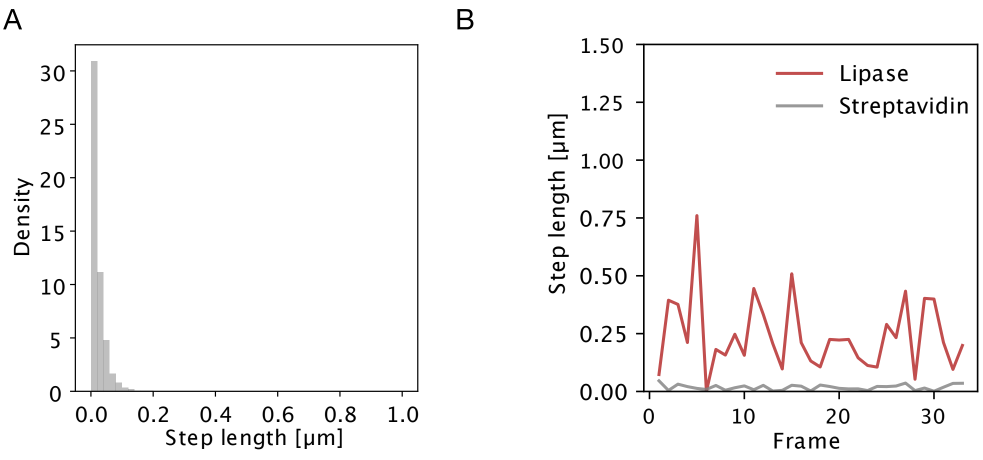

Fig. S7. Mobility of immobile streptavidin irreversibly bound to the surface via streptavidin-biotin linker)[4]. A) Distribution of step lengths for immobile proteins, practically yields the lower limit for the setup. B) Time trajectories for immobile proteins (black) and a freely diffusion lipase (red).

S8. Evaluation of anomalous diffusion parameter for all variants and conditions

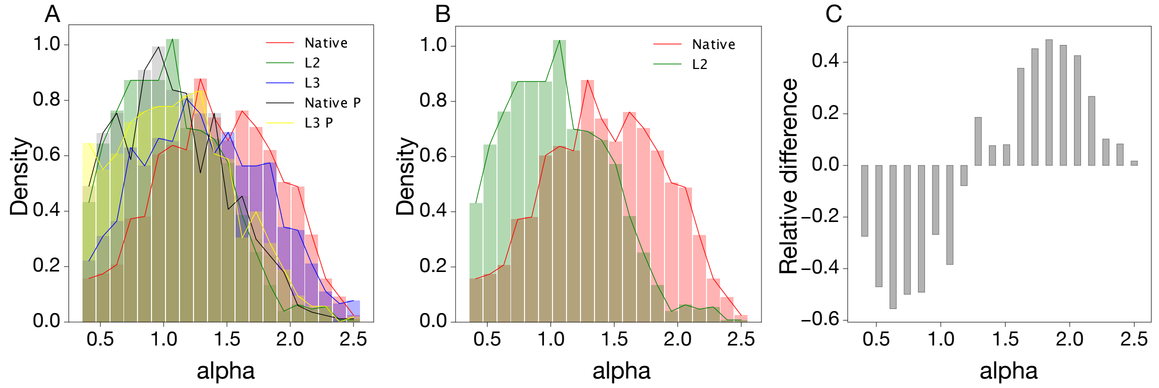

Fig. S8. Anomalous diffusion parameter. A) Anomalous diffusion parameter for all lipase mutants. Inspection reveals, that highly active variants display an increased tendency for values significantly above 1 – in agreement with our hypothesis and recently suggested models[10]. B) Direct comparison of mutant L2 and the native variant. C) Subtraction of values from B clearly visualizes the differences between highly active and less variants.

S9. Transition Density Plots for all variants and conditions

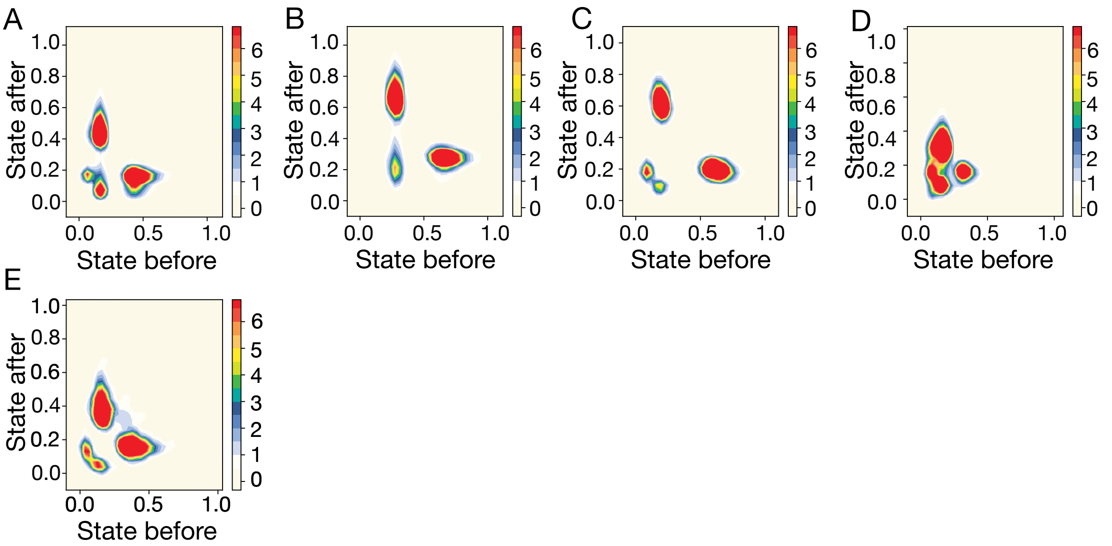

Fig. S9. Transition density plots (TDPs) displaying the frequency of transitions between states. Initial state and final state step length values for each transition are accumulated into 2D histograms for all variants and conditions. All observed transitions between states for lipase mutants reveal a linear model for shifts between states, and enabled formulation of the model. A) Mutant variant L2. B) Mutant variant L3. Note that the immobile state is sampled only to a minute degree, however visible in histograms (see Fig. 2). C) Native variant. D) Native variant under product inhibited conditions. E) Variant L3 under product inhibited conditions. We note the slight overlap of cluster for panel D, which may result in increased errors for this species, as discussed in supplementary methods M2.

S10.Transition rates for all pairs of possible transitions for all variants and conditions

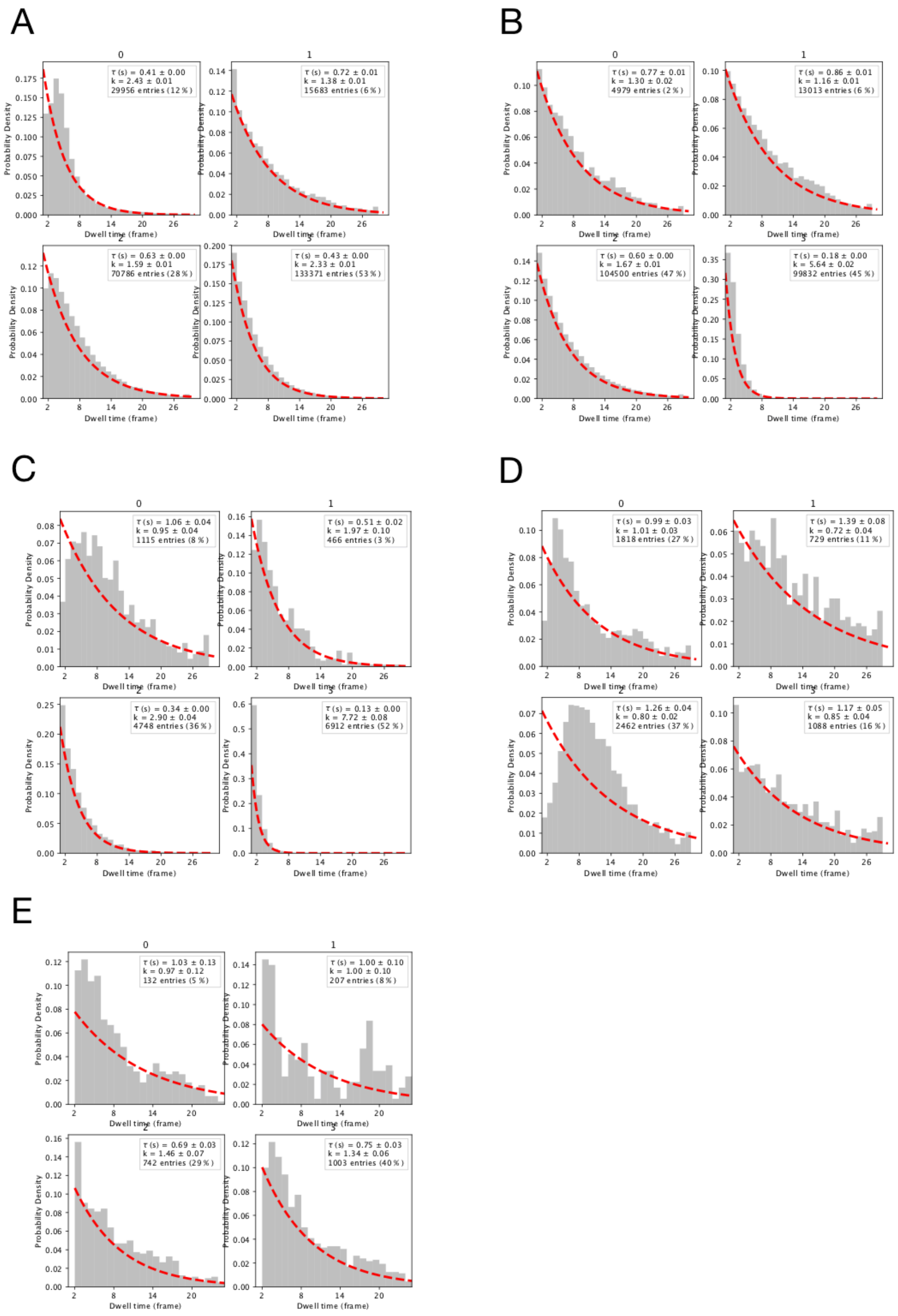

Fig. S10. Summary of lifetimes for all transitions for lipases under normal conditions, derived from TDPs using clustering (see M2). Red line indicate single exponential fit using maximum likelihood. A) Variant L2. B) Variant L3. C) Native lipase variant. D) Native product inhibited. E) L3 product inhibited. We note slightly improper fit for panel D (bottom left), which could be originate from poorly separated data in the corresponding TDP (see Fig. S9D). As for panel E (top right), we ascribe this to lack of data points. However, as 18/20 data set seems to fit well using a single exponential fit, we believe this to be the best approximation for explaining the observed behaviors.

S11. Diffusion coefficient from HMM found states

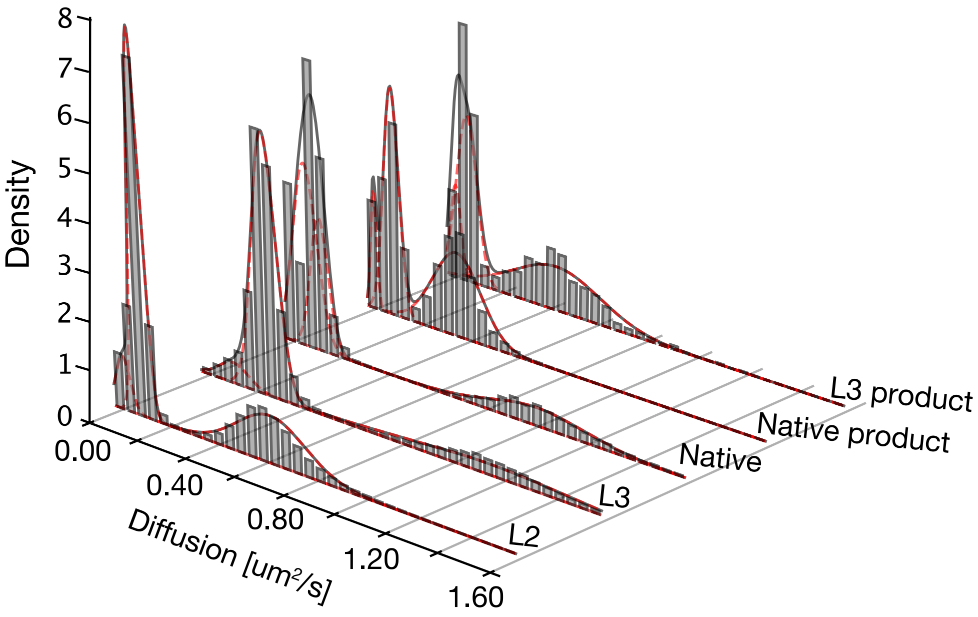

Fig. S11. Diffusion coefficients from individual HMM found states. For each trajectory each individual state were fitted to a Brownian diffusion model as described earlier. Dashed red lines indicate a gaussian fit by 3 independent distributions, black line indicate the sum of all gaussians.

S12. Model with microscopic rates for all variant and conditions

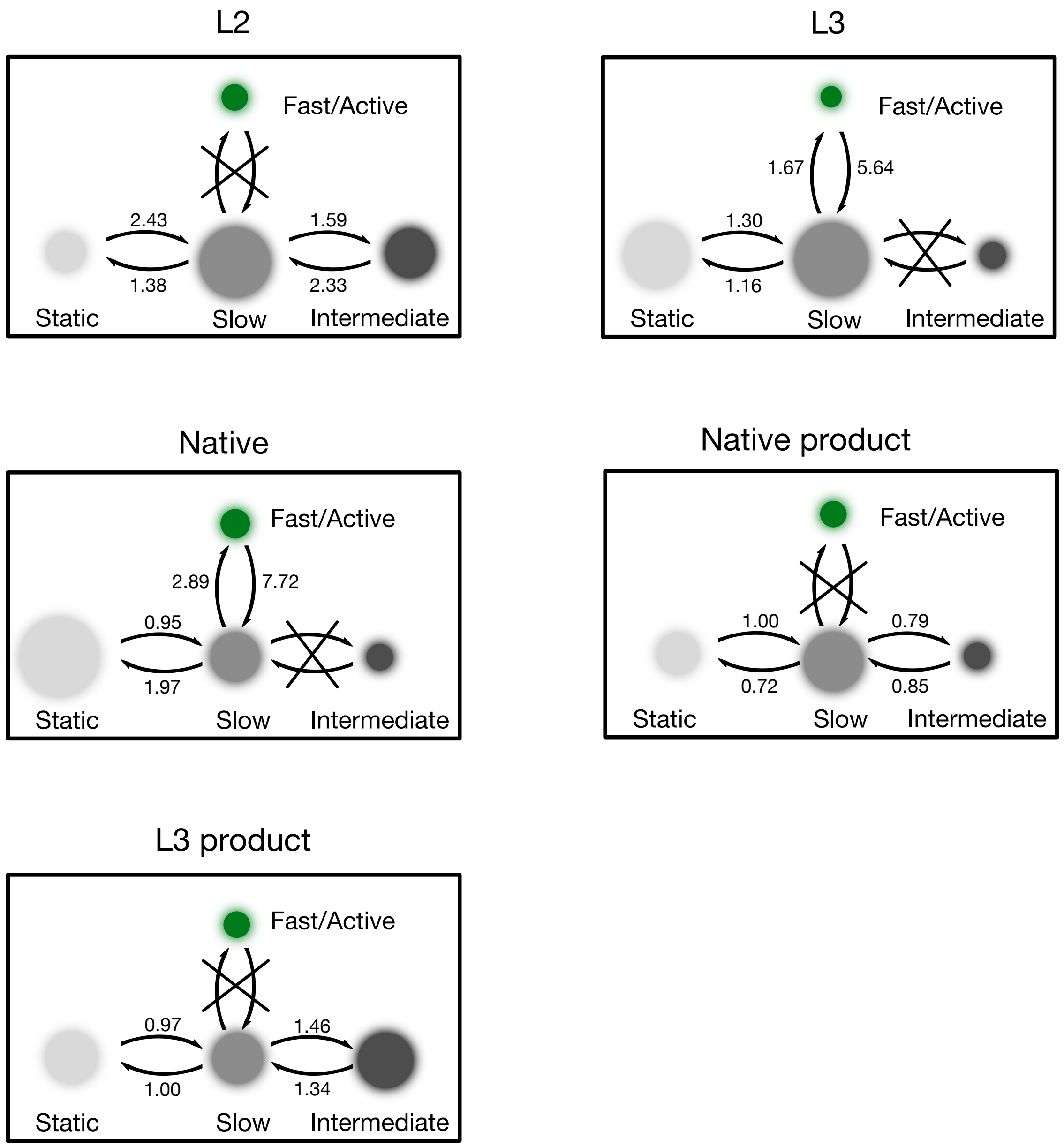

Fig. S12. Model with microscopic rates for all variants and conditions. Product inhibition operates via prohibiting transition to the fast diffusing highly active state

S13. Comparison of product inhibited variants

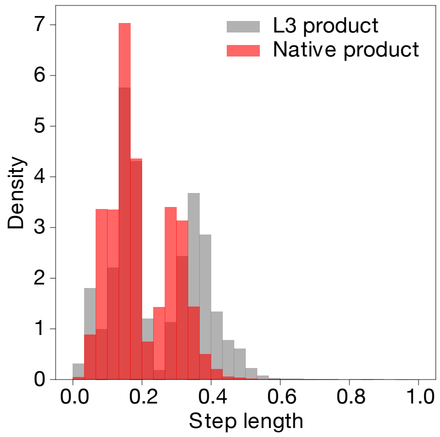

Fig. S13. Direct comparison of the idealized step lengths found via HMM for product inhibited experiments. Inspection reveals a clear tendency for L3 to move faster as expected. This is in agreement with the observed diminished inhibition for bulk activity.

Supplementary Table 1. BIC values of HMM models

**LIPASE**	**BIC VALUES FROM HMM MODELS**			
	1 component	2 components	3 components	4 components
**L2**	-2.01	-2.73	-3.28	-3.10
**L3**	-0.22	-2.09	-2.58	-1.23
**NATIVE**	-1.91	-2.46	-2.55	-1.85
**NATIVE PRODUCT**	0.01	-0.56	-0.67	0.03
**L3 PRODUCT**	0.38	-0.61	-1.21	-0.52

Supplementary Table 1. Table of BIC values from HMM analysis using gamma distributions to describe the data. For all variants, a model using 3 components is slightly preferred.

Supplementary Table 2. Activation energies and free energy difference

	Transition rates [s^{-1}]					
Lipase	k_1	k_{-1}	k_2	k_{-2}	k_3	k_{-3}
L2	70.78 ± 0.014	72.19 ± 0.015	71.84 ± 0.011	70.90 ± 0.011		
L3	72.34 ± 0.04	72.61 ± 0.028			71.71 ± 0.008	68.70 ± 0.007
Native	73.12 ± 0.10	71.31 ± 0.12			70.35 ± 0.035	67.92 ± 0.027
Native product	72.97 ± 0.08	73.80 ± 0.13	73.55 ± 0.07	73.38 ± 0.11		
L3 product	73.07 ± 0.31	72.97 ± 0.25	72.06 ± 0.011	72.26 ± 0.10		

* Error indicate one standard deviation

Supplementary Table 2. Activation energies between lipase states

Supplementary Table 3. Free energy difference

	Relative free energy			
Lipase	Immobile	Slow	Intermediate	Fast/active
L2	1.40 ± 0.2	0	0.84 ± 0.02	
L3	0.28 ± 0.05	0		3.02 ± 0.01
Native	- 1.81 ± 0.16	0		2.43 ± 0.04
Native product	0.81 ± 0.14	0	0.18 ± 0.13	
L3 product	- 0.1 ± 0.05	0	- 0.2 ± 0.06	

* Error indicate one standard deviation

Supplementary Table 3. Activation energies between lipase states

Supplementary Table 4. Statistical comparison of average lipase diffusion

	Native	L3	L2	Native product	L3 product
Native					
L3	<0.0001				
L2	<0.0001	<0.0001			
Native product	<0.0001	<0.0001	<0.0001		
L3 product	<0.0001	<0.0001	<0.0001	<0.0001	
DOPE-ATTO655	<0.0001	<0.0001	<0.0001	<0.0001	<0.0001

Supplementary Table 4. Statistical comparison of average diffusion coefficient for all tested conditions by two-sided Welch's test (assuming unequal population variance). Reported values are the two-tailed p-value for each set of comparisons having identical means.

Supplementary Table 5. Hydrodynamic radius by Stokes-Einstein

	R [nm]*
Native	1.6 ± 0.8
L3	1.2 ± 0.4
L2	1.8 ± 0.6

** Error indicate one standard deviation of at least 6 different experiments (surface preparations).*

Supplementary Table 5. Lipase hydrodynamic radius from average diffusion.

References:

1 Lin, W.-C. *et al.* H-Ras forms dimers on membrane surfaces via a protein–protein interface. *Proc. Natl. Acad. Sci. U. S. A.* **111**, 2996 (2014).

2 Thompson, C. D. K., Sharma, A. K., Frank, J., Gonzalez, R. L., Jr. & Chowdhury, D. Quantitative Connection between Ensemble Thermodynamics and Single-Molecule Kinetics: A Case Study Using Cryogenic Electron Microscopy and Single-Molecule Fluorescence Resonance Energy Transfer Investigations of the Ribosome. *J. Phys. Chem. B* **119**, 10888-10901, doi:10.1021/jp5128805 (2015).

3 Stella, S. *et al.* Conformational Activation Promotes CRISPR-Cas12a Catalysis and Resetting of the Endonuclease Activity. *Cell* **175**, 1856-1871.e1821, doi:https://doi.org/10.1016/j.cell.2018.10.045 (2018).

4 Stella, S. *et al.* Conformational Activation Promotes CRISPR-Cas12a Catalysis and Resetting of the Endonuclease Activity.

5 Kass, R. E. & Raftery, A. E. Bayes Factors. *Journal of the American Statistical Association* **90**, 773-795, doi:10.1080/01621459.1995.10476572 (1995).

6 Einstein, A. Über die von der molekularkinetischen Theorie der Wärme geforderte Bewegung von in ruhenden Flüssigkeiten suspendierten Teilchen. *Annalen der Physik* **322**, 549-560, doi:10.1002/andp.19053220806 (1905).

7 Valeri, D. & Meirelles, A. J. A. Viscosities of fatty acids, triglycerides, and their binary mixtures. *J. Am. Oil Chem. Soc.* **74**, 1221-1226, doi:10.1007/s11746-997-0048-6 (1997).

8 Ferrario, V. & Pleiss, J. Simulation of protein diffusion: a sensitive probe of protein–solvent interactions. *J. Biomol. Struct. Dyn.* **37**, 1534-1544, doi:10.1080/07391102.2018.1461689 (2019).

9 Marsh, D. Molecular volumes of phospholipids and glycolipids in membranes. *Chem. Phys. Lipids* **163**, 667-677, doi:https://doi.org/10.1016/j.chemphyslip.2010.06.005 (2010).

10 Jee, A.-Y., Dutta, S., Cho, Y.-K., Tlusty, T. & Granick, S. Enzyme leaps fuel antichemotaxis. *Proc. Natl. Acad. Sci. U. S. A.* **115**, 14, doi:10.1073/pnas.1717844115 (2018).
